# High-dose interleukin 2 in patients with metastatic renal cell carcinoma with sarcomatoid features

**DOI:** 10.1371/journal.pone.0190084

**Published:** 2017-12-20

**Authors:** Tala Achkar, Ananth Arjunan, Hong Wang, Melissa Saul, Diwakar Davar, Leonard J. Appleman, David Friedland, Rahul A. Parikh

**Affiliations:** 1 University of Pittsburgh, Department of Medicine, Division of Hematology/Oncology, Pittsburgh, Pennsylvania, United States of America; 2 University of Pittsburgh, Department of Public Health, Biostatistics, Pittsburgh, Pennsylvania, United States of America; 3 University of Pittsburgh, Department of Health Information Management, Pittsburgh, Pennsylvania, United States of America; 4 University of Pittsburgh Medical Center, Hillman Cancer Center, Pittsburgh, Pennsylvania, United States of America; 5 University of Kansas Medical Center, Westwood, Kansas, United States of America; National Institute of Health, UNITED STATES

## Abstract

**Background:**

High-dose interleukin-2 (HD IL-2) is used in the treatment of metastatic renal cell carcinoma (mRCC) and has an overall response rate (ORR) of 12–20% and a complete response rate (CR) of 8% in unselected populations with predominantly clear cell type renal cell carcinoma. Nearly 10–15% of patients with renal cell carcinoma exhibit sarcomatoid differentiation, a feature which correlates with a median overall survival (OS) of 9 months and overall poor prognosis. We report a single institution experience with 21 patients with mRCC with sarcomatoid features post-nephrectomy who were treated with HD IL-2.

**Methods:**

Twenty one patients with mRCC with sarcomatoid features post-nephrectomy who underwent therapy with HD IL-2 were identified at the University of Pittsburgh Medical Center from 2004 to 2016. Baseline patient characteristics, HD IL-2 cycles, time to progression, and subsequent therapies were evaluated. OS and progression-free survival (PFS) in the cohort were calculated using the Kaplan-Meier method. Disease characteristics were evaluated for significance using the Fischer′s exact test and Wilcoxon rank sum test.

**Results:**

Patients were predominantly Caucasian males with a median age of 54 years. A majority, 86% of these patients, had metastatic disease at time of initial presentation, primarily with lung and lymph node involvement. The ORR and CR with HD IL-2 was 10% and 5%, respectively. Initial localized disease presentation is the only variable that was significantly associated with response to HD IL-2 (p = 0.0158). Number of HD IL-2 doses did not correlate with response with a mean of 16.5 and 15.0 total doses in responders and non-responders, respectively (p = 0.53). Median PFS with HD IL-2 was 7.9 months (95% CI, 5.0–21.3). Median OS was 30.5 months (95% CI 13.3–57.66). Within the subset of patients who had progression on IL-2, median OS was 19.4 months (95% CI, 13.3–35.3). In patients who received second-line therapy, median PFS was 7.9 months (95% CI 2.4–10.2).

**Conclusion:**

In patients with mRCC with sarcomatoid features, use of HD IL-2 was associated with a modest ORR and a higher survival compared to historical controls (patients with mRCC and sarcomatoid features). Thus, HD IL-2 may have a role in treating selected patients with mRCC with sarcomatoid features.

## Introduction

Renal cell carcinomas (RCC) are classified into histological subtypes with the clear cell subtype representing 75–85% of all RCC. Sarcomatoid RCC is a distinct subtype that is defined by highly pleomorphic spindle cells along with cells that are typical of RCC. The reported incidence of the sarcomatoid type is between 0.7% to 13.2% of all renal carcinomas [1, 2]. The sarcomatoid subtype is clinically more aggressive, presents more commonly as metastatic disease, and is associated with a worse prognosis than other pathologic subtypes of RCC [1]. It is characterized by a relatively high incidence of lung and bone metastases at presentation [3]. The median overall survival (OS) for patients with sarcomatoid differentiation is 3 to 10 months from diagnosis based on prior studies [2, 3]. A higher proportion of sarcomatoid differentiation in the sample is generally associated with worse survival.

The use of high-dose interleukin-2 (HD IL-2) for RCC was introduced in the mid-1980s. Based on animal models, a regimen was developed whereby an intravenous (IV) infusion is administered every 8 hours for a maximum of 14 doses. It was evaluated in a phase II trial of 255 patients over 21 institutions [4]. The median OS for these patients was 16.3 months, but notably this was in the era prior to tyrosine kinase inhibitors (TKIs) [4]. The objective overall response rate (ORR) was 14% (CI: 10%-19%) with 5% complete response rate (CR) and a 9% partial response rate (PR) [4]. The median duration of the partial responses (PR) was 25.3 months, and the median duration of the CRs was not reached [5]. In a subsequent phase III study of HD IL-2 the ORR was as high as 23.2%, with 8.4% CRs [6].

Prognostic factors that are associated with a response to HD IL-2 include a low Memorial Sloan Kettering Cancer Center (MSKCC) score, higher baseline weight, and a lack of prior immunotherapy. There is also a weak trend toward higher response rate in patients who have had a nephrectomy [7]. Sarcomatoid differentiation has been shown to be associated with an 82% increased risk of cancer-specific death, with each 10% increase in the amount of sarcomatoid differentiation associated with a 7% increased risk of death from RCC [8]. The University of California Los Angeles (UCLA) developed a unique algorithm to predict patient survival after nephrectomy and HD IL-2 therapy called Survival After Nephrectomy and Interleukin-2 Immunotherapy (SANI). The authors incorporated the presence of sarcomatoid features as an important poor-risk variable in the UCLA SANI score. The twenty patients with sarcomatoid features had an approximately two-fold higher risk of death despite surgical resection and HD IL-2 therapy compared to patients with clear cell histology [9].

The HD IL-2 “SELECT” Trial is a prospective trial conducted by the Cytokine Working Group (CWG) that evaluated whether a predictive model could determine responsiveness to HD IL-2 in patients with metastatic RCC (mRCC) [10]. The MSKCC risk score was not found to be associated with ORR. The UCLA SANI score accounts for sites of metastatic disease, the presence of sarcomatoid features and TSH level. In patients with a high UCLA SANI score, there were no objective responses to HD IL-2, and there was a significantly worse PFS. However, this study does not specifically detail the response of patients with sarcomatoid histology to HD IL-2, although these features are incorporated into the UCLA SANI score.

Patients with mRCC with sarcomatoid features also respond poorly to VEGF-targeted therapy, which is usually used for the first-line therapy of clear cell type RCC. A retrospective study that consisted of 43 patients with metastatic sarcomatoid RCC who received VEGF-targeted therapy showed that progression free survival (PFS) was much longer in patients who had non-sarcomatoid RCC compared to those with sarcomatoid RCC (16.3 months vs 6.2 months; p< 0.001) [3]. The ORR was 50% in those with non-sarcomatoid RCC compared to 25% in those with sarcomatoid RCC (p = 0.02) [3]. Responses were only seen in those patients who had <20% sarcomatoid differentiation [3]. Studies utilizing HD IL-2 have been extensively published, however, there have been few series that have specifically evaluated the impact of sarcomatoid differentiation on response to HD IL-2. Usually, these studies have lumped patients with sarcomatoid features into a bigger non-clear cell type histology, which also includes patients with papillary and collecting duct carcinomas. Overall, there is no standard of care for the management of the sarcomatoid variant of RCC, and it is managed in an identical fashion to clear cell RCC.

In this retrospective study, we evaluate the response rates of patients with metastatic RCC and sarcomatoid differentiation to treatment with HD IL-2.

## Materials and methods

Patients with metastatic renal cell carcinoma who were treated with HD IL-2 at the University of Pittsburgh Medical Center from June 1994 to December 2016. The University of Pittsburgh Institutional Review Board (IRB) reviewed and approved this study under the designation of minimal risk (IRB#: PRO16070593). The patients’ pathology reports were retrospectively reviewed in order to identify patients with sarcomatoid differentiation. A total of 21 such patients were identified. Information, whenever available, was collected on demographics, as well as disease characteristics including sites of metastatic disease, and MSKCC score. Treatment information was also collected including the number of cycles of HD IL-2, responses, and prior and subsequent lines of treatment.

Overall survival was defined as the time from date of metastatic disease to date of last follow up or death. PFS on HD IL-2 was calculated based on the date of development of metastatic disease to the date of progression after HD IL-2, or the date of the last follow-up if the patient did not have progression. PFS to second line treatment was calculated based on the date of progression on HD IL-2 to the date of progression on second line therapy, or the date of the last follow-up if the patient did not have progression. The survival data were analyzed with the Kaplan Meier methods. Median survival and its 95% confidence interval was calculated.

The association of response to HD IL-2 with other variables was studied with the two-sided two sample t-test. The association of response to second line therapy with other variables was studied with the same method. The association of survival endpoints with other variables was analyzed with the univariate Cox proportional hazards model.

All statistical analyses were performed using **SAS version 9.4** (**SAS** Institute Inc. Cary, NC).

## Results

A total of 147 patients with metastatic renal cell carcinoma who were treated with HD IL-2 at the University of Pittsburgh Medical Center between June 1994 and December 2016 were identified. Of these, 21 patients were identified as having sarcomatoid RCC and were treated with HD IL-2 between March 2005 and December 2016.

The patients included were predominantly male (67%). All were Caucasian with a median age of 54 years. A majority (86%) of patients presented with metastatic disease, and all the patients had a nephrectomy at the time of diagnosis. The most common site of metastatic disease at presentation was the lungs (87%), lymph nodes (43%), followed by adrenal and hepatic metastases (14% each). More than half the patients (57%) were smokers, and 81% of the patients had not received any systemic treatment modalities prior to HD IL-2 (Table 1). Two patients had received sorafenib prior to HD IL-2, and one patient had received sorafenib with the addition of procarbazine, CCNU, and vincristine on a clinical study. The fourth patient had received pazopanib and pembrolizumab on a clinical study prior to treatment with HD IL-2. Only this fourth patient received subsequent treatment following HD IL-2. The patient went on to receive cabozantinib and responded to this for 9 months prior to progressing. The patient is currently responding to a combination of lenvatinib and everolimus.

**Table 1:** Patient characteristics (N = 21).

Characteristic		Number
Sex	F	7 (33%)
	M	14 (67%)
Race	white	21 (100%)
Age (at dx)	Mean	54.1
	Median	54.0
	Standard deviation	6.5
	Range	45.0–66.0
Laterality (L/R)	Left	8 (38%)
	Right	13 (62%)
Presentation (local/systemic)	Local	3 (14%)
	Systemic	18 (86%)
Nephrectomy	No	0 (0%)
	Yes	21 (100%)
Metastases at the time of Nephrectomy (Y/N)	No	1 (5%)
Site of metastatic disease	Yes	20 (95%)
Adrenal	No	18 (86%)
	Yes	3 (14%)
Lung	No	3 (14%)
	Yes	18 (86%)
Liver	No	18 (86%)
	Yes	3 (14%)
LN	No	12 (57%)
	Yes	9 (43%)
Bone	No	20 (95%)
	Yes	1 (5%)
CNS	No	20 (95%)
	Yes	1 (5%)
MSKCC Score	0 (Good)	1 (5%)
	1–2 (Intermediate)	16 (80%)
	≥3 (High)	3 (15%)
	Mean	1.6
	Median	1.0
	Standard deviation	0.8
	Range	0–3.0
Smoking (y/n)	No	9 (43%)
	Yes	12 (57%)
Tx Before IL-2	None	17 (81%)
	Pembro+Pazopanib trial	1 (5%)
	Sorafenib	2 (10%)
	Sorafenib, PCV	1 (5%)
% Sarcomatoid Features	Mean	17% (N = 10)
	Unknown	11 (52%)
	<20%	7 (33%)
	≥20%	3 (14%)
	Median	10%
	Standard deviation	20.5%

One of these patients discontinued HD IL-2 after the first cycle and did not have a documented response to treatment, so the patient was excluded from further analysis.

The ORR was 10% (90% CI: 2%-28%), with a 5% CR (90% CI: 0%-22%) and a 5% PR (90% CI: 0%-22%). Among the patients who responded to HD IL-2, the mean number of doses received was 16.2, while in the patients who did not respond, the mean was 15. The total number of doses did not have a significant effect on response, with a *p-*value of 0.5275 by Wilcoxon rank-sum test (Table 2). Of all the variables collected, only presentation with localized disease had a significant association with response to treatment (p = 0.0158; Table 2). This is consistent with time from diagnosis to treatment of greater than one year, which is a part of both MSKCC and Heng risk criteria to evaluate prognosis in this patient population.

**Table 2:** Variables associated with response to HD IL-2.

	Responders to HD IL-2	Non-responders to HD IL-2	p-value
Sex (F)	1/2 (50%)	6/18 (33%)	1.0000 with Fisher’s exact test
Age (at dx)	Mean±SD: 55.0±14.1 (n = 2)	Mean±SD: 54.1±6.1 (n = 18)	0.9497 with Wilcoxon rank sum test
Presentation (local)	2/2 (100%)	1/18 (6%)	0.0158 with Fisher’s exact test
MSKCC Score	Mean±SD: 0.5±0.7 (n = 2)	Mean±SD: 1.7±0.8 (n = 17)	0.0734 with Wilcoxon rank sum test
Smoker	2/2 (100%)	9/18 (50%)	0.4789 with Fisher’s exact test

**Fig 1:**
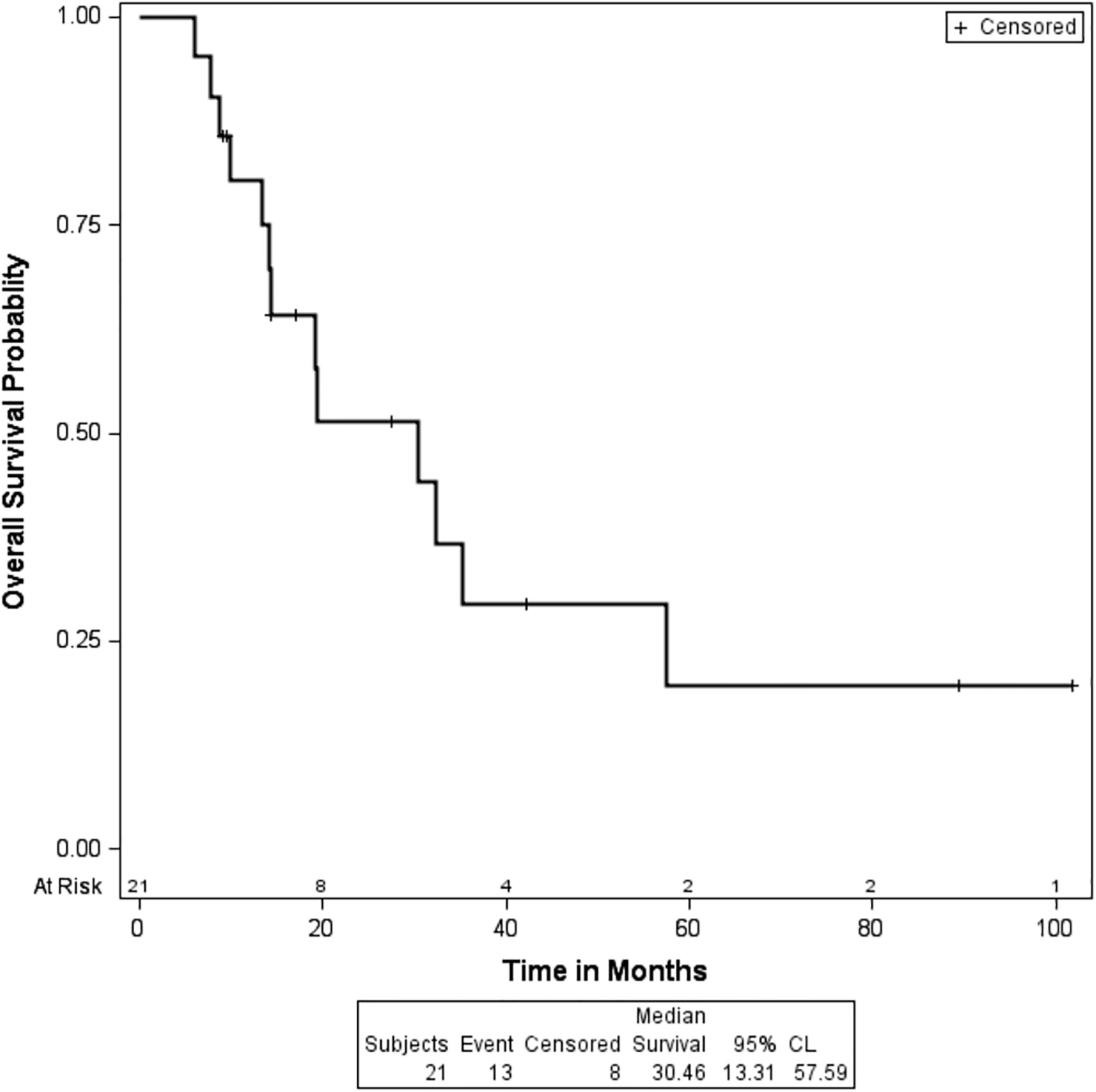
Kaplan-Meier survival curve showing the overall survival of the entire patient cohort.

Following progression on HD IL-2, 15% of patients responded to second line treatment. Second line treatment was with sunitinib in 61% of patients, pazopanib in 11%, sorafenib in 5%, and axitinib in 5%. In our study population, 62% of the patients died and the median OS was 30.5 months (95% CI: 13.3, 57.66), as depicted in Fig 1. Among the patients who did not respond to HD IL-2, 72% of the patients died with a median OS of 19.4 months (95% CI: 13.3, 35.3).

The median PFS after treatment with HD IL-2 was 7.9 months (95% CI: 5, 21.3). In the patients who received second line treatment, the median PFS with TKIs was 7.9 months (95% CI: 2.4, 10.2).

## Discussion

This paper describes the largest retrospective review of patients with sarcomatoid RCC who have been treated with HD IL-2. Sarcomatoid RCC has a worse prognosis than the other subtypes of RCC, and the prognosis is inversely correlated to the proportion of sarcomatoid histology in the tumor.

In a large review of 2000 patients with RCC, the incidence of a sarcomatoid subtype RCC was 5%. Survival rates at 2-years for patients with clear cell, papillary, and chromophobe RCC with a sarcomatoid component was 32.9%, 40%, and 27.8% respectively compared to 82.1%, 94.6% and 95.3% in those histologic subtypes without a sarcomatoid component [11]. Median OS in patients with sarcomatoid features in spite of aggressive surgery and systemic therapy was approximately 8 months [11].

Prior characterization of histologic features that are associated with tumor response to IL-2, showed that in patients with conventional renal cell carcinoma 21% (30/146 patients) respond to IL-2, whereas in non-clear cell subtypes, only 6% (1/17 patients) respond to IL-2 [12]. Granular features were associated with a significantly poorer response to IL-2. The presence and proportion of rhabdoid and sarcomatoid subtypes did not show an association with response to IL-2 [12]. The presence of sarcomatoid components in clear cell RCC, chromophobe RCC, and papillary RCC was associated with a statistically significant increased risk of death [13].

The HD IL-2 “SELECT” evaluated whether the UCLA SANI score, a predictive model which accounts for sarcomatoid histology, could determine responsiveness to HD IL-2 in patients with mRCC [10]. They found that in patients with a high UCLA SANI score, there were no objective responses to HD IL-2, and there was a significantly worse PFS. However, this study does not specifically detail the response of patients with sarcomatoid histology to HD IL-2, although these features are incorporated into the UCLA SANI score. Also, only five of 120 patients had non-clear cell histology of various types [10]. This is also seen in other studies, where patients with sarcomatoid histology have been combined with other non-clear cell variants in statistical analysis. In a small retrospective review of 31 patients with sarcomatoid histology, 9 patients received HD IL-2 [1]. In this patient group, the median survival had not been reached with a median follow up of 10.4 months. 2 patients had partial responses, and no complete responses were seen. There was no correlation between response and percentage of sarcomatoid tumor.

In the largest cohort of patients with sarcomatoid histology, 2,286 patients were analyzed retrospectively [14]. The time from original diagnosis to relapse in sarcomatoid patients was 18.8 months compared to 42.9 months in patients without sarcomatoid features. 21% of patients with sarcomatoid RCC responded to treatment with VEGF inhibitors compared with 26% of patients with non-sarcomatoid RCC. The median OS for sarcomatoid patients was 10.4 months compared to 22.5 months in non-sarcomatoid patients [14].

In order to evaluate the response of patients with sarcomatoid features to therapeutic options, a phase II study of sunitinib and gemcitabine in sarcomatoid and poor risk patients with mRCC was completed [15]. There were a total of 72 patients, and the ORR was 26% for patients with sarcomatoid RCC. Median OS was 10 months for patients with sarcomatoid RCC. There are also a number of clinical trials underway, as described in Table 3.

**Table 3:** Ongoing clinical trials evaluating treatment options for sarcomatoid RCC.

Clinical Trial	Description
NCT01767636	Phase II Efficacy Trial of Pazopanib in Non-clear Cell Metastatic Renal Cell Cancer
NCT01164228	Randomized Phase II Trial of Sunitinib and Gemcitabine or Sunitinib Alone in Advanced Renal Cell Carcinoma with Sarcomatoid Features
NCT00126503	A Phase I/II Trial of Sorafenib with Bevacizumab in Patients with Advanced Renal Cancer

In concordance with the literature we found that the highest incidence of metastases in our patient population was lung metastases seen in nearly 87% of patients studied. In our population, however, the median OS in patients who did not respond to HD IL-2 was 19.4 months, which is double the OS described in the literature [2, 3]. This may be confounded by the fact that new treatment options have been made recently available including the checkpoint inhibitors as well as the TKIs, cabozantinib and lenvatinib.

The ORR to HD IL-2 was 10%, with a 5% CR and a 5% PR which is similar to the rates previously reported. Unfortunately given the small number of patients, we are unable to determine if there is a higher response rate and duration of response to VEGF-TKIs following treatment with HD IL-2.

In the “SELECT” study, they evaluated tumors for programmed death-ligand 1 (PD-L1) expression by immunohistochemistry [10]. Eighteen of the tumors were determined to express PD-L1, although it is not reported which histological subtype these tumors belonged to. They found that response to HD IL-2 correlated positively with tumor expression of PD-L1 (p = 0.01), and that durable remissions also correlated with PD-L1 expression (p < 0.01). PD-L1 expression has been evaluated in RCC with and without sarcomatoid differentiation. In a published series, PD-L1 expression was identified in 17% of clear cell RCC, and 54% of RCC with sarcomatoid differentiation [16]. Interestingly, it has been suggested that tumors that have expression of PD-L1 are less likely to respond to VEGF-TKIs. Checkpoint blockade has shown promising results in the treatment of mRCC, and given that sarcomatoid tumors have high expression of PD-L1 this suggests that the responses may be seen in patients with sarcomatoid histology.

Limited evidence exists for the use of checkpoint inhibitors in the sarcomatoid subtype of RCC. The late-phase studies with nivolumab excluded patients with non-clear cell type histology [17, 18]. In a phase I study with atezolizumab, the ORR for patients with Fuhrman grade 4 disease or sarcomatoid features (n = 18) was an impressive 22%. However, the number of patients with and extent of sarcomatoid differentiation was not reported [19]. There have been case reports of patients with RCC with sarcomatoid features with a rapid response to nivolumab [20]. However, more studies need to be performed to evaluate the response of checkpoint inhibitors in non-clear cell type histology. Checkpoint inhibitors and HD IL-2 are associated with a high financial burden. With HD IL-2, this cost is usually related to inpatient stay requiring specialized care, as well as potential intensive care unit management secondary to toxicities. With the use of checkpoint inhibitors, the cost is composed of the agent, as well as the duration of therapy, the extent of which has not yet been clearly defined in prospective studies.

Our study was limited by the overall number of patients, as well as its retrospective nature which led to some missing data. Importantly, our study provides some insight that despite the worse prognosis that sarcomatoid differentiation confers, these patients do still respond to HD IL-2 treatment. HD IL-2 should remain an option for otherwise healthy, young patients with excellent performance status who have sarcomatoid RCC.
